# Transfection of *clMagR/clCry4* imparts MR-T_2_ imaging contrast properties to living organisms (*E. coli*) in the presence of Fe^3+^ by endogenous formation of iron oxide nanoparticles

**DOI:** 10.3389/fmolb.2023.1119356

**Published:** 2023-02-17

**Authors:** Nuan Li, Le Xue, Xiaoli Mai, Peng Wang, Chenzhuo Zhu, Xiaofeng Han, Yuanyuan Xie, Bin Wang, Yuqing Ge, Yewei Zhang, Jianfei Sun

**Affiliations:** ^1^ State Key Laboratory of Bioelectronics, School of Biological Sciences and Medical Engineering, Southeast University, Nanjing, China; ^2^ Jiangsu Key Laboratory of Biomaterials and Devices, Southeast University, Nanjing, China; ^3^ Department of Radiology, The Affiliated Drum Tower Hospital of Nanjing University, Nanjing, China; ^4^ Department of Sports Medicine and Adult Reconstructive Surgery, The Affiliated Drum Tower Hospital of Nanjing University, Nanjing, China; ^5^ Southeast University-Monash University Joint Graduate School, Southeast University, Suzhou, China; ^6^ Clinical Stem Cell Center, The Affiliated Drum Tower Hospital of Nanjing University, Nanjing, China; ^7^ State Key Laboratory of Transducer Technology, Shanghai Institute of Microsystem and Information Technology, Chinese Academy of Sciences, Shanghai, China; ^8^ The Hepatopancreatobiliary Center, The Second Affiliated Hospital of Nanjing Medical University, Nanjing, China

**Keywords:** contrast agent, iron oxide nanoparticles, biosynthesis, MRI, *clMagR/clCry4*

## Abstract

Rapid development of medical imaging, such as cellular tracking, has increased the demand for “live” contrast agents. This study provides the first experimental evidence demonstrating that transfection of the *clMagR/clCry4* gene can impart magnetic resonance imaging (MRI) T_2_-contrast properties to living prokaryotic *Escherichia coli* (*E. coli*) in the presence of Fe^3+^ through the endogenous formation of iron oxide nanoparticles. The transfected *clMagR/clCry4* gene markedly promoted uptake of exogenous iron by *E. coli*, achieving an intracellular co-precipitation condition and formation of iron oxide nanoparticles. This study will stimulate further exploration of the biological applications of *clMagR/clCry4* in imaging studies.

## Introduction

Contrast-enhanced magnetic resonance imaging (MRI) has become an indispensable tool in medical imaging (Rogers et al., 2006; Park et al., 2017). Gadolinium (Gd)-based small molecules and iron oxide nanoparticles have been mainly used as MRI-T_1_ and -T_2_ contrast agents, respectively, to provide high contrast sensitivity against background signals and have been approved by the U.S. Food and Drug Administration (FDA) and the European Medicines Agency (EMEA) (Park et al., 2017; Wahsner et al., 2019). Nevertheless, there are still some challenges facing these contrast agents in practical applications, especially for long-term tracking *in vivo*. The main issue lies in their biosafety and the exocytosis of inorganic substances over a long period (Wahsner et al., 2019). For example, Gd was recently reported to be somewhat toxic to the liver and kidney (Calvin et al., 2010), and iron (Fe) overload has also been proposed to be involved in degenerative diseases (Zecca et al., 2004; Stephenson et al., 2014). Thus, it has inspired a new wave to develop new contrast agents, where control of iron accumulation in cells could work as a means to alter longitudinal or transverse relaxation times, generating contrast, probably derived endogenously from the organism itself.

Recently, protein-based MRI contrast agents have attracted increasing attention, such as transferrin and ferritin (Jutz et al., 2015; Schilling et al., 2017). Transferrin and ferritin are critical proteins that regulate iron metabolism *in vivo*, which are capable of binding Fe to exhibit paramagnetic properties (Yang et al., 2016). Although the two proteins, especially ferritin, have relatively large magnetic moments and influence the transverse relaxation of the proton, the effect appears to be too weak to be used directly as contrast agents for MRI *in vivo* (Liu and Theil, 2005; Jutz et al., 2015). Generally, ferritin is used as a natural reactor *in vitro* to transform hydrated paramagnetic ferric oxide into superparamagnetic iron oxide nanoparticles (Liu and Theil, 2005; Turano et al., 2010). These transformed protein molecules have been proposed as a new type of nanoparticle and partly improve the biosafety of contrast agents; however, they are essentially an exogenous substance.

Xie et al. discovered a novel magnetic protein biocompass in *Columba livia*, which integrated both magnetoreceptor (MagR) and type IV cryptochrome (clCry4) (Qin et al., 2016). MagR is the homologue of the iron–sulfur cluster assembly protein (A-type ISC protein, IscA). The IscA protein is an iron chaperon that can bind to intracellular iron to form Fe–S clusters for electron transfer (Ding and Clark, 2004; Holm and Lo, 2016). The Cry4 protein is considered an electron donor excited by light that can form long-lived radical pairs to activate downstream pathways (Maeda et al., 2012; Zoltowski et al., 2019; Xu et al., 2021). Because clMagR/clCry4 protein is involved with the process of electron transfer, we hypothesized it would be suitable for MRI contrast as a “live” agent.

We investigated the MRI contrast performance of *clMagR/clCry4* gene in a living organism. Unlike the strategy used for ferritin, the *clMagR/clCry4* gene was simply transfected into prokaryotic *E. coli*, rather than administering clMagR/clCry4 protein molecules. The transfected *E. coli* showed significant T_2_ contrast on MRI when cultured in an iron-supplemented medium, while the protein itself was unable to show this effect. The transfection of the *clMagR/clCry4* gene led to the formation of iron oxide nanoparticles within *E. coli*, which could mediate the alteration of the MRI transverse relaxation rate.

## Materials and methods

### MRI analysis

The MRI study was performed using the 7T BioSpec 70/20 USR system (Bruker Biospin; Ettlingen) with ParaVision 6.0.1. software. The MRI-T_1_ images were acquired using a rapid acquisition sequence with relaxation enhancement (RARE) using the following parameters: matrix = 256 × 256, flip angle (FA) = 90°, field of view (FOV) = 8.0 × 6.03 cm, slice thickness = 2 mm, echo time (TE) = 8.87 ms, and repetition time (TR) = 400 ms. The MRI-T_2_ images were acquired using the TurboRARE sequence using the following parameters: matrix = 256 × 256, FA = 90°, FOV = 8.0 × 6.03 cm, slice thickness = 2 mm, TE = 80 ms, and TR = 2,200 ms. For MRI-T_1_ relaxation time map imaging (T_1_ mapping), we used the RARE sequence with variable TR with the following parameters: matrix = 256 × 256, FA = 90°, FOV = 8.0 × 6.03 cm, slice thickness = 2 mm, TE = 7.17 ms, echo spacing = 7.17 ms, averages = 2, repetition = 1, and TR = 190, 200, 300, and 400 ms. For MRI-T_2_ relaxation time map imaging (T_2_ mapping), we used a multislice multiecho (MSME) sequence with variable TE with the following parameters: matrix = 256 × 256, FA = 90°, FOV = 8.0 × 6.03 cm, slice thickness = 2 mm, TE = 9–225 ms with an increment of 9 ms, echo spacing = 9.0 ms, echo times = 25, averages = 1, repetition = 1, and TR = 3,000 ms. The regions of interest (ROIs) were drawn in the same plane of each sample after scanning, the T_1_ and T_2_ relaxation times were calculated using ParaVision software, and the rates at which the signal decayed were defined as R_1_ and R_2_.

The Python language (Python Software Foundation, Version 3.8.0) was used to batch calculate the mean gray values and to quantify the signal density of the MRI images (Sindhulakshmi et al., 2014; Gadi et al., 2020). In detail, original DICOM images were processed with a Gaussian filter. On the basis of the maximum between-class variance method (OTSU), the image data were classified into targets and backgrounds. The OpenCV (OpenSource Computer Vision Library, Version 4.4.0) contour search algorithm was adopted to extract the true coordinate parameters of each target area and to discard false values. Furthermore, the aforementioned coordinates were processed to obtain the mean gray value of each target area pixel group on the original image, all pixels were sorted according to their mean gray values, and the average value and the quartile value were recorded. The heat map of the original gray value was drawn using Numerical Python (NumPy), Pandas, Matplotlib, and OpenCV. In this study, to facilitate the statistics of MRI dark contrast, the mean gray values mentioned included 255 (the maximum gray value of the 8-bit gray image) minus the original gray values.

The bacteria were collected by centrifugation (2,000 rpm, 5 min, 4°C), the pellets were washed three times, and resuspended in phosphate buffer solution (PBS, pH 7.4). The density of bacteria cells was evaluated by optical density (OD) measurement at 600 nm (A600 units/mL). Protein concentrations were determined using the bicinchoninic acid (BCA) assay with each sample in triplicate, using the microplate method according to the protocol recommended by the manufacturer using commercially available BSA as the calibration solution. Data were plotted in the graph form, and a linear trendline was fit to obtain a standard BSA protein curve. Data were acquired and analyzed using the SpectraMax Plus microplate reader and SoftMax Pro software (Molecular Devices). The MRI samples were collected into polyethylene centrifugation tubes for scanning.

### Intracellular iron quantification

The bacteria were collected after MRI scanning, lysed in nitric acid, and then analyzed by inductive-coupled plasma mass spectrometry (ICP-MS, PerkinElmer NexION 2000). For calibration, the reference solutions containing different concentrations of iron (i.e., 0, 20, 50, 100, 200, and 500 μg/L in Milli-Q water, 18.2 MΩ cm) as internal standards were prepared. A reference solution was used at the beginning, middle, and end of the measurements as a quality control. Acidity of experimental samples and reference solutions were controlled at 5%. Experimental samples were filtered through a 0.22-μm hydrophilic syringe filter (Sartorius Stedim Biotech, Germany) to remove solid impurities. Data corresponding to iron content were determined by ICP-MS. The iron standard curve, determined from calibration solutions with known concentrations (μg/L), was used to calculate the iron content in bacteria.

### Detection of intracellular pH

Intracellular pH was measured using the fluorescent pH indicator 2,7-bicarboxyethyl-5,6-carboxyfluorescein-acet-oxymethylester (BCECF-AM) according to the manufacturer’s protocol (Burgess and Han, 2010; Chakraborty et al., 2017). Bacterial suspensions (OD 2.0) were incubated with 20 μM BCECF-AM at 37 °C for 60 min. After loading, the cells were washed three times with PBS buffer and remained in the same solution. A pH calibration curve was constructed using BCECF-AM with a pH calibration buffer kit containing a pH range of 4.5, 5.5, 6.5, and 7.5, and valinomycin (10 μM) and nigericin (10 μM), which equilibrated the intracellular and extracellular pH of bacteria. Intracellular pH was recorded by determining the fluorescence ratio (F490 nm/F440 nm) of the emission wavelength at 535 nm for excitation wavelengths of 490 and 440 nm using a multimode microplate reader (Tecan Infinite M200). Bacterial photographs were detected using inverted fluorescence microscopy (Nikon Microsystems).

### Ultrastructural observation

The bacteria were collected by centrifugation, pellets washed with PBS and then fixed with 2.5% glutaraldehyde overnight at 4°C. The samples were fixed with 1% osmium tetroxide for 1 h, dehydrated with a series of ethanol concentrations in Milli-Q water (i.e., 35, 50, 60, 70, 80, 90, 95, and 100% ethanol for 10 min in each step) and then embedded in epoxy resin and polymerized at 60°C overnight. Ultrathin (about 90 nm) sections were cut with a diamond knife in an ultramicrotome (Leica, EM UC7) and collected onto carbon-coated copper grids, stained with uranyl acetate and lead citrate, and then examined by transmission electron microscopy (TEM) (Hitachi H-600-4) at an operating voltage of 120 KV.

### Biomaterial extraction and electronic microscope analysis

To extract electron-dense granules, *clMagR/clCry4*-transfected bacteria (under exogenous iron supply condition) were harvested, washed, and resuspended in PBS and then fragmented using an ultrasonic cell disruptor (Scientz-II D, amplitude 15%, pulse 5 s on and 2 s off). Subsequently, electron-dense granules were separated from cell debris using gradient centrifugation, washed and resuspended in Milli-Q water to remove residual PBS. The biosynthesis of materials was isolated from the suspension using the MACS LD separation column, the QuadroMACS Separator, and the MACS MultiStand (Miltenyi Biotec Inc) according to the manufacturer’s instructions. The resulting materials were placed on carbon-coated copper grids and dried. Bright-field scanning transmission electron microscopy (BF-STEM), dark-field STEM (DF-STEM), high-angle annular dark-field STEM (HAADF-STEM), energy-dispersive X-ray spectroscopy (XEDS) element mapping, selected area electron diffraction (SAED), and high-resolution TEM (HRTEM) were carried out on FEI Talos F200X TEM at an operating voltage of 200 KV. All micrographs were analyzed using DigitalMicrograph software (Gatan Microscopy Suite, Version 3.42.3048.0) and ICDD PDF-4 + 2009 software (The International Centre for Diffraction Data, ICDD; Powder Diffraction File, PDF). The fast Fourier transform (FFT) was performed using ImageJ, v1.53a software.

### Statistical analysis

GraphPad Prism software (v8.3.1 (332), La Jolla, CA, United States) was used for graph preparation. Data were presented as mean ± standard deviation (SD). Statistical differences were analyzed using the unpaired Student’s t-test. For mean gray values, statistical differences were conducted using IBM SPSS Statistics 25 software packages and analyzed using two-way analysis of variance (ANOVA) followed by the Bonferroni and Tukey’s honest significant differences (HSD) *post-hoc* test. The differences were considered statistically significant when the *p-*value was less than 0.05. *p < 0.05, **p < 0.01, ***p < 0.001; no significance (ns), *p* > 0.05.

## Results and discussion

**FIGURE 1:**
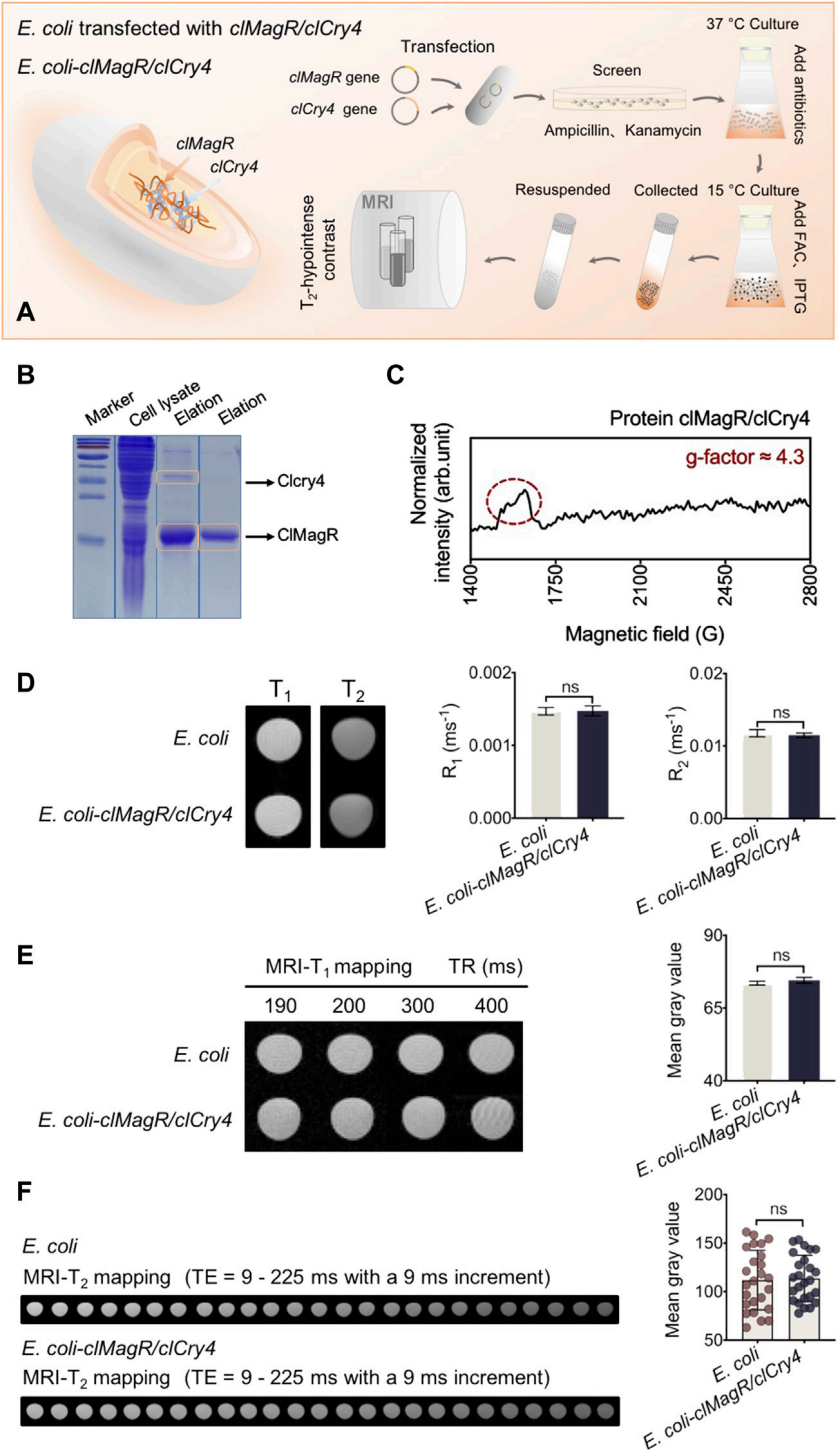
Bacteria construction and MRI analysis. **(A)** Schematic illustration of the prokaryotic expression system and bacterial culture. *clMagR/clCry4* is transfected into a commonly used bacterial model (as heterologous host), the *E. coli* BL21 (DE3) strain to investigate MRI susceptibility. **(B)** SDS-PAGE analysis. Arrows point to clMagR and clCry4. PageRuler prestained protein ladder was used to indicate the apparent molecular mass standards (marker, kDa). **(C)** ESR spectrum of the clMagR/clCry4 protein. Signal at g' ≈ 4.3 in spectrum attributed to protein-Fe(III) complex. **(D)** MRI-T_1_ and -T_2_ images show the signal intensity of *E. coli*. Histograms represent bacterial R_1_ and R_2_ statistical values. **(E)** Bacterial MRI-T_1_ mapping and the corresponding mean gray values analysis. **(F)** Bacterial MRI-T_2_ mapping and the corresponding mean gray values analysis. It should be noted that the bacterial densities of samples were almost equivalent (A600 units/mL, OD 50.0). Data were presented as mean ± SD (*n* = 3). For MRI R_1_ and R_2_ values, statistical differences were analyzed using the unpaired Student’s t-test. For the mean gray value data, statistical significances were analyzed using two-way ANOVA followed by the Bonferroni and Tukey’s HSD *post-hoc* test. ns, no significance (*p* > 0.05).

Transfection of the *clMagR/clCry4* gene into *E. coli* is shown schematically in Figure 1A. Similar to that of our previous report (Xue et al., 2020), transfection was shown to be successful based on the sodium dodecyl sulfate polyacrylamide gel electrophoresis (SDS-PAGE) pattern of the expressed clMagR/clCry4 protein (Figure 1B). Because the *d* electrons in Fe atom have been demonstrated to contribute to the net spins of the Fe-loaded IscA monomer (Beinert et al., 1997; Xiao et al., 2020), the characteristic signal at g' ≈ 4.3 (g-factor) in the electronic spin resonance (ESR) spectrum proved the presence of the protein–Fe(III) complex (Figure 1C) (Sun and Chasteen, 1994; Wajnberg et al., 2018). Furthermore, the broad peak of the ESR also indicated the heterogeneity of the protein clMagR/clCry4 protein expressed inside *E. coli*. The bacteria were resuspended in PBS buffer solution for cell suspensions (Supplementary Figure S1). Then, the *clMagR/clCry4*-transfected *E. coli* were tested by qualitative MRI-T_1_ and -T_2_ scans. As shown from the images, there were no significant differences for either mode nor were there any differences in the relaxation rates R_1_ and R_2_ (Figure 1D). Furthermore, MRI-T_1_ and -T_2_ mapping modes were used to quantitatively evaluate the imaging contrast of transfected *E. coli*. The mean gray values acquired from the mapping images were measured using the Python language and two-way ANOVA statistics, followed by the Bonferroni and Tukey’s HSD *post-hoc* test. Both the images and the statistical values of the mean gray areas also showed minimal differences (Figures 1E, F). In addition, only the clMagR-transfected *E. coli* also showed a little imaging contrast with the control (Supplementary Figure S2). We tested the MRI contrast properties for the purified clMagR/clCry4 protein. The protein itself showed a little MRI contrast effect (Supplementary Figure S3). Based on these cases, the transfection of the *clMagR/clCry4* gene was unable to influence MRI spin relaxation signals. Due to its presence in a living organism, the clMagR/clCry4 protein could perceive a magnetic field; thus, we hypothesized that the magnetism of the clMagR/clCry4 protein would associate with the electron transfer, that is, binding with Fe. Because there was insufficient Fe in the medium for *E. coli*, the *clMagR/clCry4* complex was incapable of producing magnetic behaviors.

**FIGURE 2:**
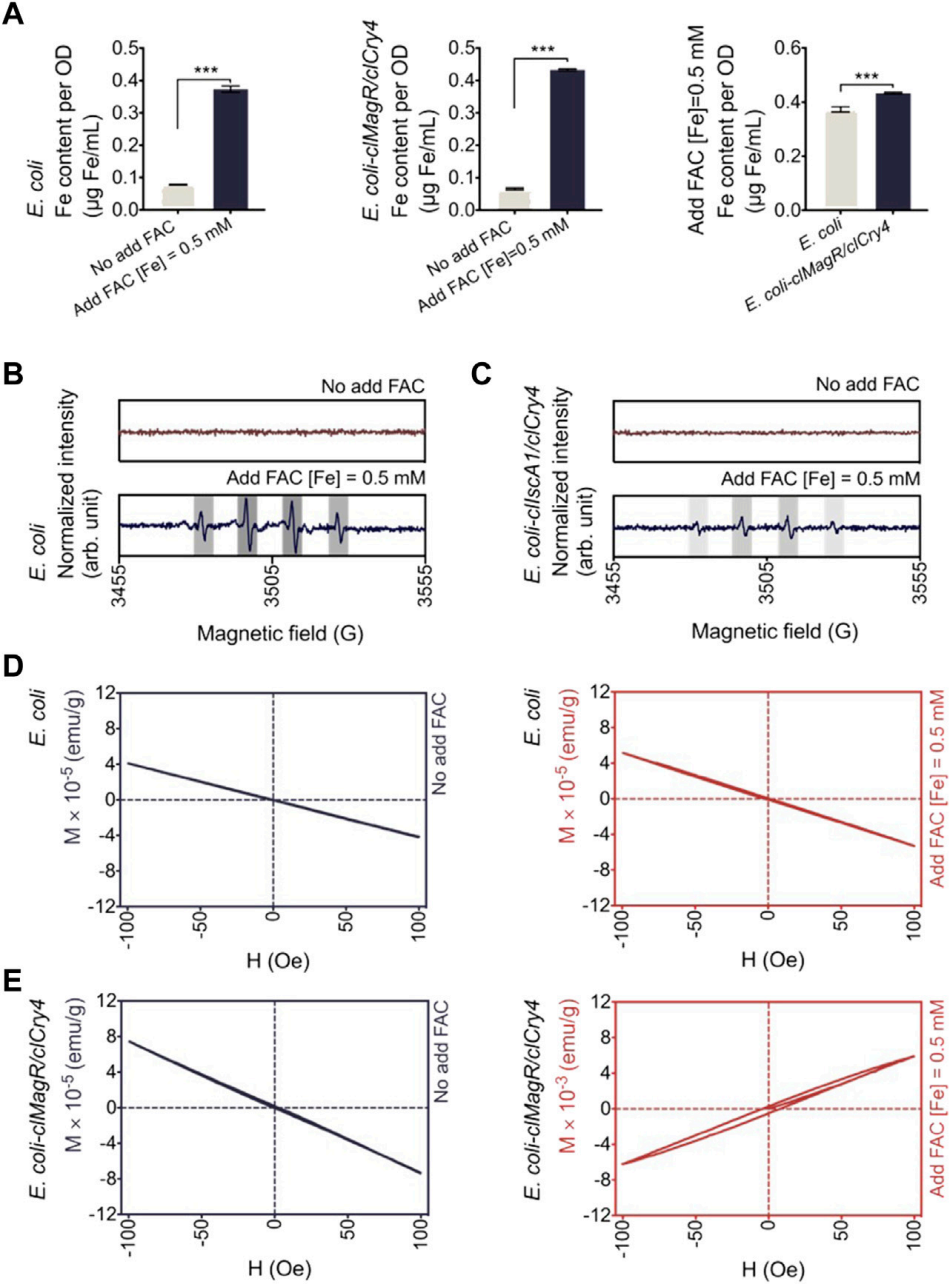
Biological effects of exogenous iron on bacteria. **(A)** Iron content analysis. Data corresponding to iron content were determined by ICP-MS. The intracellular Fe content of *E. coli* transfected with *clMagR/clCry4* was higher than that of the control. Data were presented as mean ± SD (*n* = 3). Statistical significance was analyzed using the unpaired Student’s t-test. ***p < 0.001. **(B,C)** Hydroxyl radical generation was monitored by ESR measurement. The main quartet signal characteristic for the •DMPO-OH adduct is indicated by gray columns. Compared to control *E. coli*, the quartet signal was also detected at by *E. coli* transfected with *clMagR/clCry4* but at a much lower intensity. Data were normalized for intergroup difference comparison. **(D,E)** Hysteresis loops (magnetization M versus applied field H) of *E. coli* were measured by SQUID magnetometry. When cultured in the exogenous iron supply medium, *E. coli* transfected with *clMagR/clCry4* showed detectable magnetic properties (magnetic susceptibility, 0.00625 emu/g).

Thus, we added ammonium ferric citrate (FAC) to the medium as an exogenous iron donor. FAC is a soluble ferric salt that has been approved by the U.S. FDA as a food supplement and clinical drug (Tenne et al., 2015). Hence, it is safe and has clear effects, as confirmed by scanning electron microscopy (SEM) and live/dead BacLight stain tests. As revealed by the SEM micrographs, *E. coli* retained its typical rod-shaped morphology without any discernible alteration (Supplementary Figure S4). The fluorescence pattern of SYTO 9 and double-staining with propidium iodide (PI) further confirmed the good viability of bacteria as shown in Supplementary Figure S5, where green indicates living cells and red indicates dead cells. Beyond that, the addition of FAC significantly increased the intracellular iron content within bacteria, and *E. coli* transfected with *clMagR/clCry4* gene showed higher levels than the control, which was experimentally verified by ICP-MS (Figure 2A). However, the status of iron within the bacteria was different. Dissociative Fe will produce abundant hydroxyl free radicals resulting from the Fenton reaction, while Fe binding could greatly reduce this production of free radicals (Dixon and Stockwell, 2014). Thus, we used ESR to identify the hydroxyl free radicals with the spin adduct of 5,5-dimethyl-1-pyrroline N-oxide (DMPO)-hydroxyl radicals. For control *E. coli*, the quartet signal was unambiguously detected, indicating the presence of abundant hydroxyl free radicals (Figure 2B). However, for *E. coli* transfected with *clMagR/clCry4* gene, the intensity of the corresponding ESR signal decreased greatly, meaning that dissociated Fe was bound (Figure 2C). A similar case occurred for *E. coli* transfected with *clMagR* gene, while the intensity decrease was slightly lower than that of *E. coli* transfected with *clMagR/clCry4* gene (Supplementary Figure S6). Thus, we assumed that the dissociative Fe within *E. coli* was bound to the clMagR/clCry4 protein, causing the alteration of bacterial magnetism. The SQUID measurement confirmed this point. As predicted, control *E. coli* were diamagnetic, even after exposure to FAC (Figure 2D). A similar diamagnetic curve was also observed in *E. coli* transfected with *clMagR/clCry4* gene in the absence of an exogenous iron supply. However, the bacteria exhibited a paramagnetic curve after culture in the presence of media containing an exogenous iron supply, and the intensity of magnetization increased by two orders of magnitude (Figure 2E). As shown by the magnetization loop, there was even a very slight hysteresis, which seemed somewhat superparamagnetic (magnetic susceptibility, 0.00625 emu/g). Surprisingly, this effect was not observed in *clMagR*-transfected *E. coli*, where the bacteria retained the diamagnetic behavior (Supplementary Figure S7). Therefore, these findings supported the potential effects on MRI contrast of transfection with *clMagR/clCry4* gene in the presence of exogenous iron supply.

**FIGURE 3:**
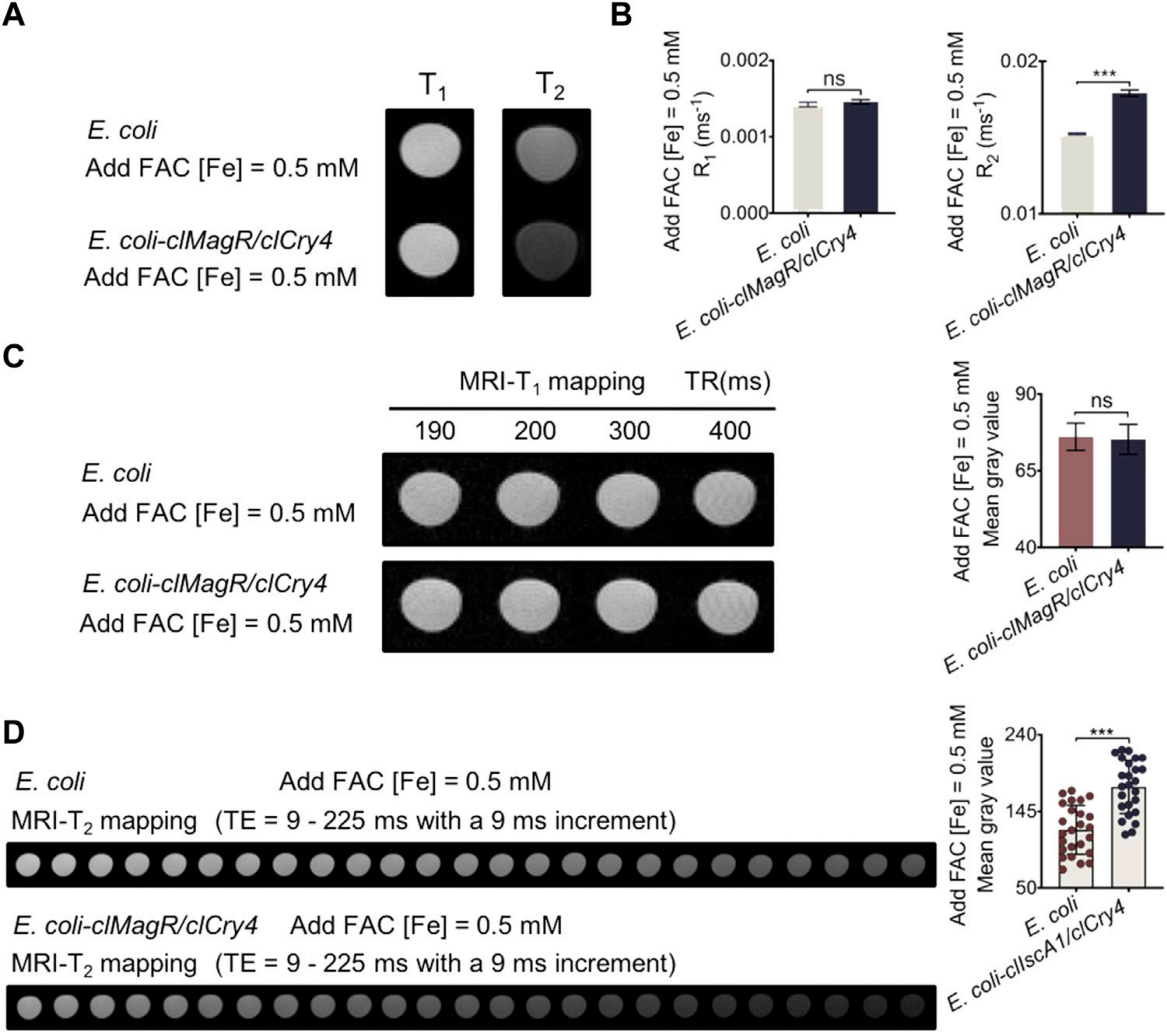
MRI analysis of bacteria under exogenous iron supply conditions. **(A)** MRI-T_1_ and -T_2_ images showing signal intensity of bacteria. **(B)** MRI statistical R_1_ and R_2_ values analysis of the bacteria. **(C)** Bacterial MRI-T_1_ mapping and the corresponding mean gray values analysis. **(D)** Bacterial MRI-T_2_ mapping and the corresponding mean gray values analysis. It should be noted that the bacterial densities of samples were almost equivalent (A600 units/mL, OD 50.0). Data were presented as mean ± SD (*n* = 3). For MRI R_1_ and R_2_ values, statistical differences were analyzed using the unpaired Student’s t-test. For the mean gray value data, statistical significances were analyzed using two-way ANOVA followed by Bonferroni and Tukey’s HSD *post-hoc* test. ns, no significance (*p* > 0.05), ***p < 0.001.

As shown by the qualitative MRI scan, the *clMagR/clCry4*-transfected *E. coli* exhibited significant MRI-T_2_ contrast after culturing with FAC, while there was little influence on the MRI-T_1_ mode (Figure 3A); the histograms represent the corresponding statistical R_1_ and R_2_ values (Figure 3B). Furthermore, to quantitatively confirm the imaging contrast effect, the mean gray values acquired from the mapping of MRI-T_1_ and -T_2_ images (Figures 3C, D) were calculated. Independently of the time of TE, it can be clearly seen that *clMagR/clCry4* gene transfection imparted the contrast effect of MRI-T_2_ to *E. coli*, which was significantly different from that of the control *E. coli* strains. Moreover, the influence of the FAC concentration (exogenous iron level) in the medium, and the bacterial density on the imaging contrast effect, was evaluated (Supplementary Figures S8, S9). As described in detail, with an increase in the iron level or bacterial density assayed, clMagR/clCry4 protein achieved a more significant influence on the T_2_-contrast of the transfected *E. coli*. Here, the corresponding original gray values were plotted against the TE for different bacterial densities after pseudocolor processing (Supplementary Figure S10). With an increase in the bacterial density, the MRI gray value of *E. coli-*transfected *clMagR/clCry4* gene tended to go from a constant to a high-order power function versus the TE time course of MRI-T_2_ mapping.

To explore the underlying mechanism responsible for MRI-T_2_ contrast *E. coli* transfected with *clMagR/clCry4* cultured in the presence of FAC, the purified clMagR/clCry4 protein was tested for contrast properties in the MR imaging. As shown in Supplementary Figure S11, the protein itself was unable to achieve MRI-T_2_ contrast signals. Thus, an explanation should lie with event results within living organisms. To validate this hypothesis, we first tested the intracellular pH of *E. coli* after transfection of *clMagR/clCry4* gene using the pH-sensitive organic dye BCECF-AM, which has been widely used in elaborating the physiology of prokaryotes and eukaryotes (Burgess and Han, 2010; Chakraborty et al., 2017). As shown in Supplementary Figure S12, the fluorescence alteration indicated that the microenvironment inside *E. coli* changed from a weakly alkaline pH to acidic pH with the addition of FAC. However, it was found that transfection of *clMagR/clCry4* gene inhibited this change in the pH value to maintain a weakly alkaline state, the detailed mechanism of which remains unclear. Apart from that, similar evidence was obtained by means of the conventional glass electrode pH meter measurement (Supplementary Table S1). On the other hand, the Fe ions will bind to biological molecules within the bacteria. It has been known that *E. coli* secreted the siderophore enterobactin to chelate Fe^3+^ with high affinity, forming soluble Fe (III)-siderophores to transport inside *E. coli* (Liu et al., 1993; Faraldo-Gomez and Sansom, 2003). Fe ions could then be released either by siderophore hydrolysis or by reduction of flavin oxidoreductase, after which the dissociative Fe would be recruited by the clMagR/clCry4 protein (Coves et al., 1993; Raymond et al., 2003). However, Fe-binding residues in clMagR are flexible and easily disrupted, so the Fe ions can partially dissociate into a free state resulting from the reactive oxygen species (Rouault and Klausner, 1996; Ding and Clark, 2004). In the presence of Fe ions under weakly alkaline conditions, the formation of iron oxide nanoparticles is assumed, which is somewhat similar to a co-precipitation reaction occurring within *E. coli*. Hence, it should be noted that the FAD group of the clCry4 protein is widely known to generate free radicals under photonic action, which may explain why *clMagR/clCry4* transfection is advantageous in the MRI-T_2_ contrast of *E. coli* compared to *clMagR* transfection alone.

**FIGURE 4:**
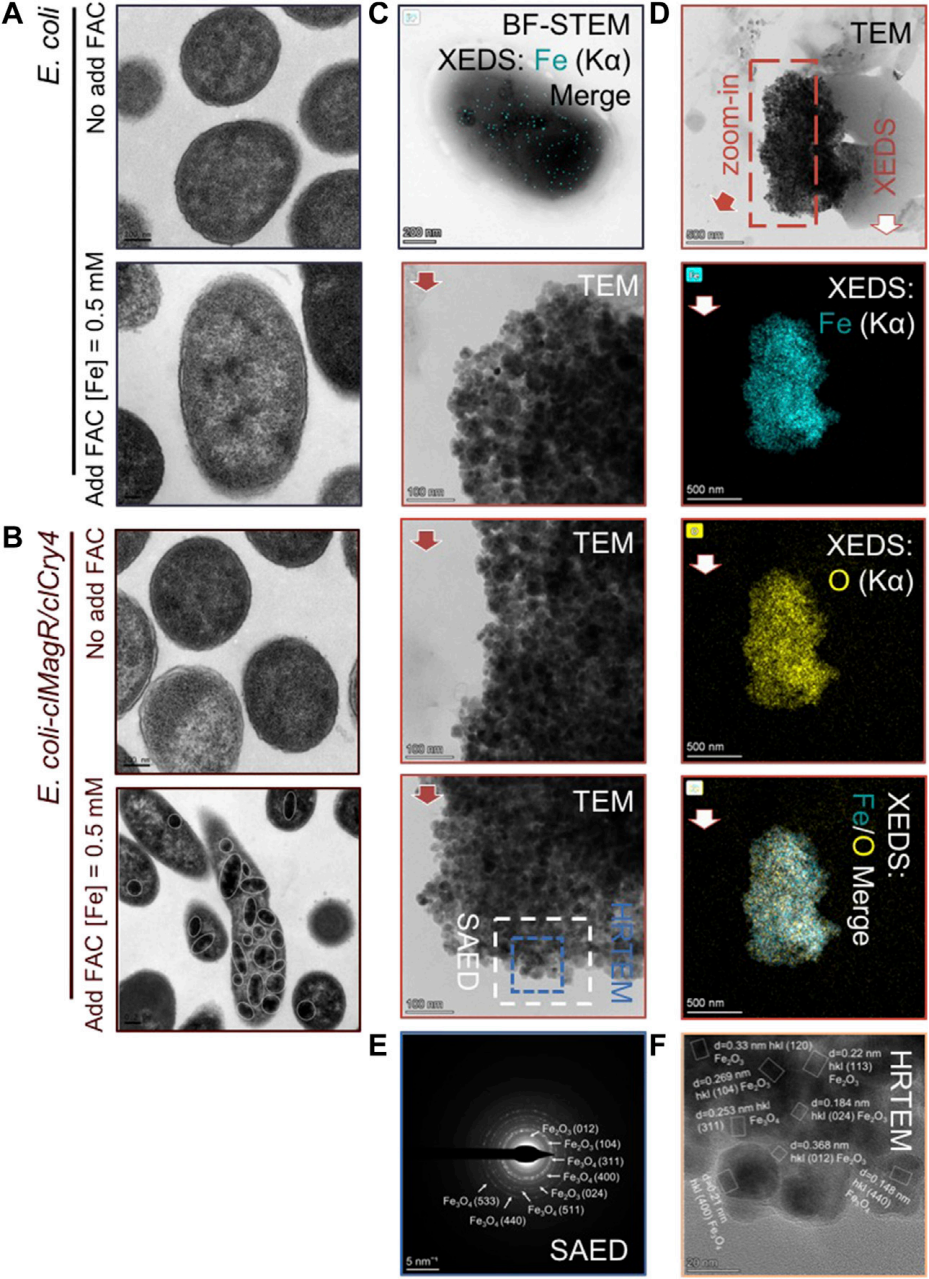
Detailed information on intracellular granules. **(A,B)** Detection of the intracellular ultrastructure. Following the transfer of *E. coli* transfected with *clMagR/clCry4* into the exogenous iron supply medium, the electron micrograph shows that electron-dense granules were detectable in the cytoplasm and form widely distributed aggregates, marked by solid circles*.* **(C)** Electron micrographs and the corresponding XEDS Fe (Kα) elemental mapping of *E. coli* transfected with *clMagR/clCry4* under exogenous iron supply conditions. **(D)** Electron micrographs and corresponding XEDS elemental mappings of the two-dimensional morphologies of the extract particles. **(E,F)** SAED and HRTEM patterns indicated by the dotted frame in **(D)**.

We used TEM to observe ultrathin bacterial sections. Under low magnification, many electron-dense granules were clearly observed in the *E. coli* transfected with *clMagR/clCry4* in the presence of exogenous iron supply (Figures 4A, B). Fe element mapping further indicated that there were abundant iron-based compounds detectable within *E. coli* (Figure 4C). Hence, we speculated that the electron-dense granules should contain the iron-based nanomaterials. These electron-dense granules were then extracted from the bacteria for TEM characterization by repeating magnetic separation and washing. Interestingly, a magnet could be used to easily attract the extracted granules (Supplementary Video S1), indicating that the magnetic iron oxide nanoparticles were present. In the TEM micrographs, aggregates of tiny nanoparticles can be clearly observed (Figure 4D). The exact matching of Fe and O elements in the XEDS mapping confirmed that the composition of the nanoparticles was iron oxide.

In addition, STEM micrographs were acquired together with XEDS elemental mappings to give complementary information on the particles being imaged, and the extracted particles demonstrated a small size range (averaged 20.62 ± 3.5 nm) (Supplementary Figure S13). Furthermore, SAED and HRTEM demonstrated the coexistence of the Fe_2_O_3_ and Fe_3_O_4_ polycrystal phases in the tiny nanoparticles (Figures 4E, F), which provides evidence supporting the presence of a magnetization loop and the MRI-T_2_ contrast effect of *clMagR/clCry4* transfected in *E. coli*. Nonetheless, it should be mentioned that aggregates of tabular-like ferric oxide nanoparticles with poor crystalline features were also detected (Supplementary Figure S14). These amorphous precursors demonstrated, in part, that the formation process of such nanoparticles was somewhat similar to that achieved by a chemical co-precipitation method.

## Conclusion

This study was the first to exploit the transfection of the *clMagR/clCry4* gene to produce an MRI-T_2_ contrast agent effect on a living organism. This innovative phenomenon has neither been observed nor hypothesized previously. Using *E. coli* as the model organism, an exogenous iron supply was shown to be a critical factor for this phenomenon. Exogenous Fe could be internalized in *E. coli* and form a dynamic “bind-release” process with the clMagR/clCry4 protein. During the cycle, a biosynthesis process occurred in the presence of dissociative Fe under weakly alkaline intracellular conditions, which is somewhat similar to the chemical co-precipitation reaction, leading to the formation of tiny iron oxide nanoparticles. These tiny iron oxide particles influence the transverse relaxation observed on MRI. Because this strategy depends on the activity of the associated protein complex, it has a wide range of potential applications that are dependent on the presence of an exogenous iron supply. We believe that this novel technique will play a key role in future biomedical imaging and tracking applications. Furthermore, due to the key role of clMagR/clCry4 protein in magnetoreception, our findings are useful for harmonizing the long-term controversy over the existence of magnetoreceptors in organisms, ranging from prokaryotes to animals.

## Data Availability

The original contributions presented in the study are included in the article/Supplementary Material; further inquiries can be directed to the corresponding author.
