# Supplementary material for: Transfection of clMagR/clCry4 imparts MR-T2 imaging contrast properties to living organisms (E. coli) in the presence of Fe3+ by endogenous formation of iron oxide nanoparticles
Source: Front Mol Biosci. 2023 Feb 17;10:1119356. doi: 10.3389/fmolb.2023.1119356 (PMC9981785; doi:10.3389/fmolb.2023.1119356)
Supplement: Supplementary file 2 [file DataSheet1.docx]

Original Article

**Transfection of *clMagR/clCry4* imparts MR-T_2_ imaging contrast properties to living organisms (*E. coli*) in the presence of Fe^3+^ by endogenous formation of iron oxide nanoparticles**

Nuan Li ^1, 2^, Le Xue ^1, 2^, Xiaoli Mai ^3^, Peng Wang ^1, 2, 4^, Chenzhuo Zhu ^5^, Xiaofeng Han ^1^, Yuanyuan Xie ^6^, Bin Wang ^6^, Yuqing Ge ^7^, Yewei Zhang ^8^, and Jianfei Sun ^1, 2*^

^*^Corresponding author E-mail: [sunzaghi@seu.edu.cn](mailto:sunzaghi@seu.edu.cn)

Table of contents

**Materials and methods**

. . ……………………………………………………………..….…………….…….4-7

Bacteria transfection and expression of heterologous protein

. . ……………………………………………………………..….…………….…….....4ESR measurement of protein iron-binding activity

Bacteria morphology characterization

Live/Dead stain

. . ……………………………………………………………..….…………….…….....5

Determination of hydroxyl radical generation by ESR

Magnetic measurement

. . …………………………………………………………..….…………….………....6

Detection of intracellular pH

. . …………………………………………………………..….…………….………....7

Supplementary Figure

. . ……………………………………………………………..….…………………8-23

1. Schematic illustration of the bacterial sample.

. . ……………………………………………………………..….…………….…….....8

1. MRI analysis of the *E. coli* transfected with *clMagR*.

. . ……………………………………………………………..….…………….…….....9

1. MRI analysis of the clMagR/clCry4 protein.

. ……………………………………………………………..….…………….…..…...10

1. SEM observation.

. ……………………………………………………………..….…………….…..…...11

1. Live/Dead stain.

. . ……………………………………………………………..….…………….……...12

1. Hydroxyl radical generation monitored by ESR measurement.

. . ……………………………………………………………..….…………….……...13

1. Hysteresis loops of the *E. coli* transfected with *clMagR*.

. . ……………………………………………………………..….…………….……...14

1. MRI-T_2_ analysis of the *E. coli* transfected with *clMagR/clCry4* under exogenous iron supply conditions.

. . ……………………………………………………………..….…………….……...15

1. MRI-T_2_ analysis of the *E. coli* transfected with *clMagR/clCry4* under exogenous iron supply conditions.

. . ……………………………………………………………..….…………….……...16

1. MRI-T_2_ analysis of the *E. coli* transfected with *clMagR/clCry4* under exogenous iron supply conditions.

. . ……………………………………………………………..….…………….……...17

1. MRI-analysis of the clMagR/clCry4 protein under exogenous iron supply conditions.

. . ……………………………………………………………..….…………….……...18

1. Intracellular pH detection.

. . ……………………………………………………………..….…………….……...19

1. Electron microscope analysis.

. . ……………………………………………………………..….…………….……...20

1. Electron microscope analysis.

. . ……………………………………………………………..….…………….……...21

1. Intracellular pH detection.

. . ……………………………………………………………..….…………….……...22

1. The particles obtained in the *E. coli* transfected with *clMagR/clCry4* migrate toward the NdFeB magnet.

. . ……………………………………………………………..….…………….……...23

**Materials and methods**

**Bacteria transfection and expression of heterologous protein**

*Columba livia* *clMagR* with the Strep-II tag plasmid and *clCry4* with the His-tag plasmid were provided by Can Xie (Hefei Institutes of Physical Sciences, Chinese Academy of Sciences). Gram-negative *E. coli* were selected as the model bacteria. The plasmids were transfected into the *E. coli* strain BL21(DE3) competent for the construction of recombinant bacteria and grown on Luria-Bertani (LB) agar plates supplemented with [antibiotics](javascript:;) for screening colonies (37 °C). Competent bacteria harboring plasmid were inoculated in medium supplemented with kanamycin (50 μg/mL) to screen the *clMagR*-transfected bacteria (*E. coli-clMagR*), ampicillin (100 μg/mL) and kanamycin (50 μg/mL) were used to screen the *clMagR/clCry4-*transfected bacteria (*E. coli-clMagR/clCry4*). Positive colonies were selected under antibiotic screening and protein expression was induced by isopropyl β-D-thiogalactoside (IPTG, 20 μM). The bacteria were incubated for an additional 24 h at 15°C with continuous shaking. Following culture, bacterial suspensions were washed three times by centrifugation (6500 g for 5 min at 4 °C) and resuspended in lysis buffer (20 mM Tris, pH 8.0) with a protease inhibitor cocktail. Cell lysates were obtained by an ultrasonic cell disruptor (Scientz-II D, amplitude 10%, pulse 6 s on and 9 s off) until visually transparent. The lysate was centrifuged at 11,000 g for 60 min at 4 °C and then the soluble fraction was collected. The supernatant was combined with the HisPur Ni-NTA matrix, and then washed with (20 mM Tris, 150 mM NaCl, 20 mM imidazole, pH 8.0) to wash the matrix, followed by elution buffer (20 mM Tris, 150 mM NaCl, 300 mM imidazole, pH 8.0). The protein obtained were purified using the Strep-Tactin matrix. After matrix washing with washing buffer (20 mM Tris, 150 mM NaCl, pH 7.5), the proteins were eluted using elution buffer (20 mM Tris, 150 mM NaCl, 5 mM desthiobiotin, pH 7.5). In the interim, the Ni-NTA matrix and the Strep-Tactin matrix were equilibrated with lysis buffer. The purified proteins were subjected to sodium dodecyl sulfate polyacrylamide gel electrophoresis (SDS-PAGE) to confirm protein expression.

**ESR measurement of protein iron-binding activity**

The freshly bacteria were transferred to 4 mm thin wall quartz electron spin resonance (ESR) sample tube (Wilmad 707-SQ-250 M) under an argon inert gas atmosphere and rapidly frozen in liquid nitrogen. Spectrum was recorded at X-band on Bruker ESR-300 spectrometer using Oxford Instruments ESR-9 flow cryostat. ESR conditions: microwave frequency, 9.45 GHz; modulation frequency, 100 kHz; microwave power, 20 milliwatt; modulation amplitude, 2.0 mT; temperature, 4.5 K; receive gain, 1.0 × 10^5^.

**Bacteria morphology characterization**

Following culture, the bacteria were collected by centrifugation, pellets washed and resuspended in phosphate buffer solution (PBS, pH 7.4) at the same cell density (OD, A600 units/mL). The bacteria were fixed with 2.5 % glutaraldehyde at 4 °C, PBS buffer solution washed and then dehydrated with a series of ethanol concentrations in Milli-Q water (i.e., 35, 50, 60, 70, 80, 90, 95 and 100 % ethanol for 10 min in each step). The dehydrated samples were allowed to dry completely, sputter coated with gold (∼15 nm) with Quorum Q-150T ES Sputter Coater before view by scanning electron microscope (SEM, Hitachi Model TM-1000).

**Live/Dead stain**

Following culture, the bacteria were collected by centrifugation, pellets washed and resuspended in PBS at the same cell density. The LIVE/DEAD BacLight Viability Kit was used according to the manufacturer’s instructions, and the mixture was incubated for 15 min in dark before applying it on 35 mm glass bottom cell culture dish. The green fluorescent dye SYTO 9 binds to the nucleic acid of both healthy and compromised cell membranes. In contrast, propidium iodide (PI) enters cells with significant membrane damage, which are considered as nonviable dead, binds with higher affinity to nucleic acids, and fluoresce red. This allows the visual differentiation between viable live and nonviable dead cells. Samples were detected using confocal laser scanning microscopy (CLSM, Nikon A1R HD25).

**Determination of hydroxyl radical generation by ESR**

Following culture, the bacteria were collected by centrifugation, pellets washed and resuspended in PBS at the same cell density, pre-warmed to 37 °C, and then •5,5-dimethyl-1-pyrroline N-oxide (DMPO) nitrone spin-trapping adduct was added before transferred to 4 mm thin wall quartz sample tube (Wilmad 707-SQ-250 M). ESR spectrum was recorded at X-band on Bruker ESR A300 spectrometer. ESR conditions: microwave frequency, 9.78 GHz; modulation frequency, 100 kHz; microwave power, 20 mW; modulation amplitude, 0.1 G; scan width, 100 G; temperature 300 K; receive gain, 5×10^4^.

**Magnetic measurement**

Following culture, the bacteria were collected by centrifugation, pellets washed and resuspended in PBS at the same cell density. The Bacteria were fully freeze-dried into powders using pilot freeze dryer ([SP Scientific](https://www.yiqi.com/brand/detail_4722.html), Advantage 20EL-85). Magnetic measurements were performed on Quantum Design SQUID (Superconducting Quantum Interference Device) MPMS-XL (Magnetic Properties Measurement System) for direct current applied field ranging from -100 to 100 Oe and the magnetic hysteresis loops were recorded at temperature 300 K.

Detection of intracellular pH

The bacterial suspensions were shaken thoroughly and detected using conventional glass electrode pH meter (Mettler Toledo FE20) for pH quantitation measurement and . The pH electrode was checked and calibrated using a three-point calibration with buffer solutions (pH 4.01, 7.00, 9.21) at room temperature. It is necessary to allow the pH probe to adequately equilibrate before recording the pH value by discard the first three values.


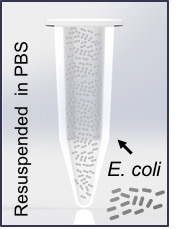


Schematic illustration of the bacterial sample. Cell suspensions were added into polyethylene centrifugation tubes and IKA Vortex1 was used to make cell density uniformity.


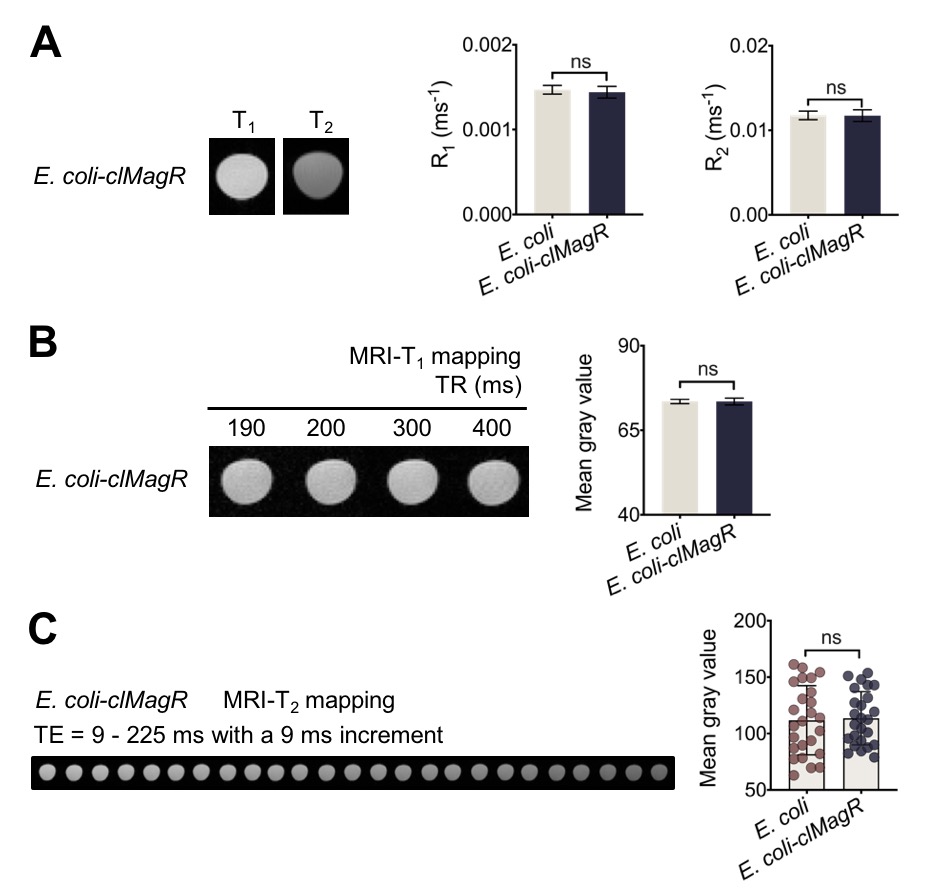


MRI analysis of the *E. coli* transfected with *clMagR*. **(A)** MRI-T_1_ and -T_2_ images show the signal intensity of bacteria. Histograms represented the statistical values of R_1_ and R_2_. **(B)** Bacterial MRI-T_1_ mapping and the corresponding mean gray values analysis. **(C)** Bacterial MRI-T_2_ mapping and the corresponding mean gray values analysis.It should be noted that the bacterial densities of samples were almost equivalent (A600 units/mL, OD 50.0). Data were presented as mean ± SD (n = 3). For MRI R_1_ and R_2_ values, statistical differences were analyzed using the unpaired Student's t-test. For the mean gray value data, statistical significances were analyzed using two-way ANOVA followed by Bonferroni and Tukey’s HSD *post-hoc* test. ns, no significance (*P* > 0.05).

**
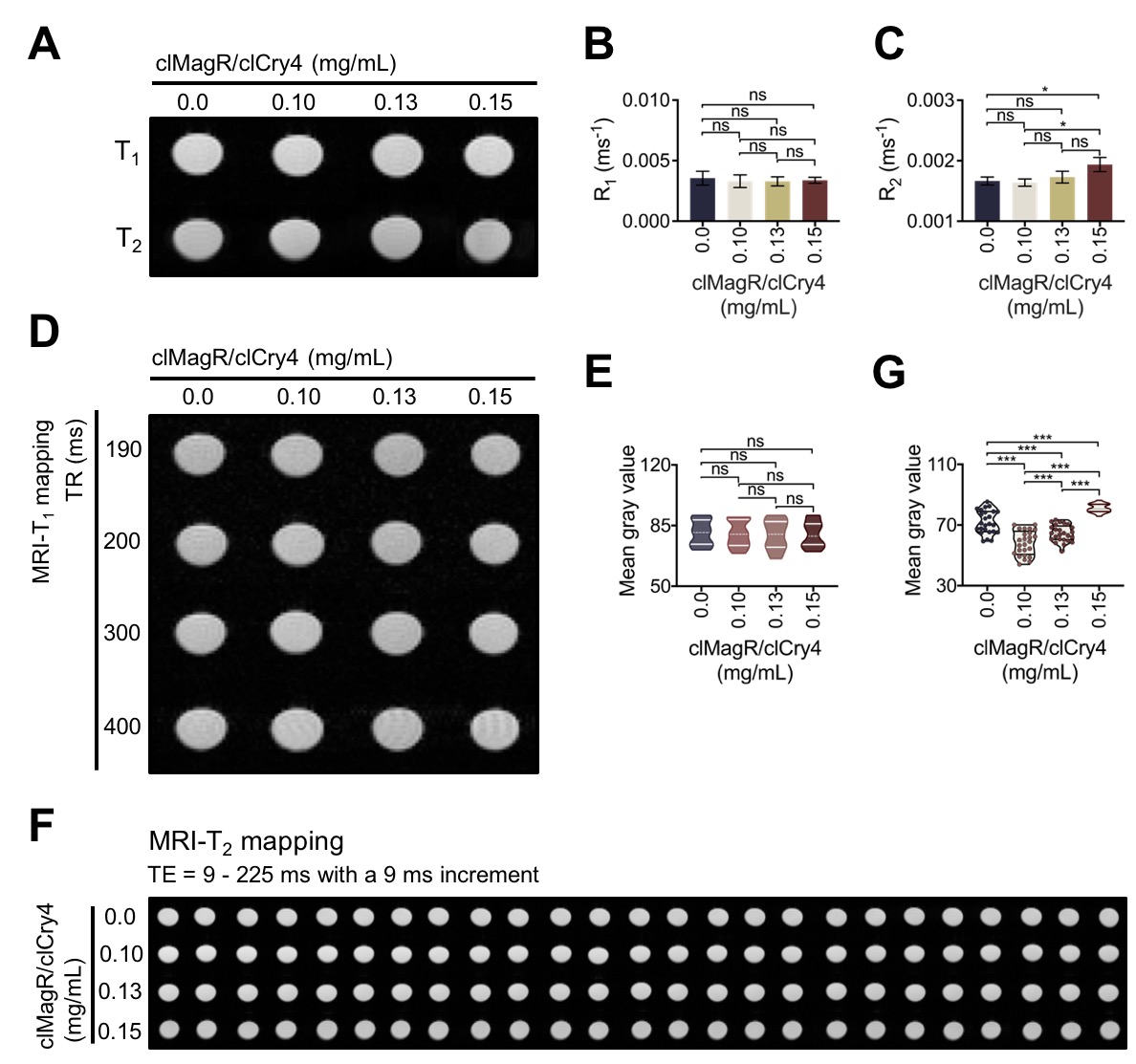
**


MRI analysis of the clMagR/clCry4 protein. **(A)** MRI-T_1_ and -T_2_ images show the signal intensity of the protein. Protein buffer solution was served as control (0 mg/mL). **(B,C)** Histogram representing the statistical values of R_1_ and R_2_. **(D,E)** Protein MRI-T_1_ mapping and the corresponding mean gray values analysis. **(F,G)** Protein MRI-T_2_ mapping and the corresponding mean gray values analysis. Data were presented as mean ± SD (n = 3). For MRI R_2_ values, statistical differences were analyzed using the unpaired Student's t*-*test. For the mean gray value data, statistical significances were analyzed using two-way ANOVA followed by Bonferroni and Tukey’s HSD *post-hoc* test. ns, no significance (*P* > 0.05), **P* < 0.05, ****P* < 0.001.


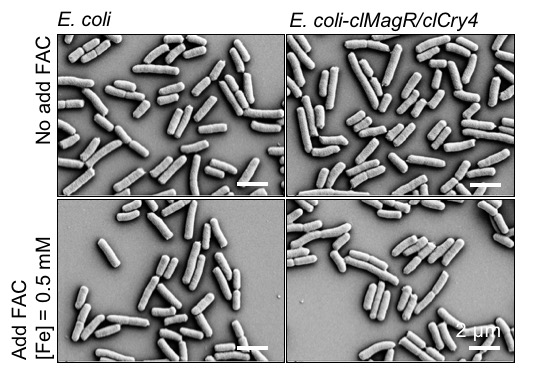


SEM observation. SEM scanning was used to visualize changes in *E. coli* morphology. The *E. coli* of the FAC tested were similar to that of the control, with no difference in morphology.


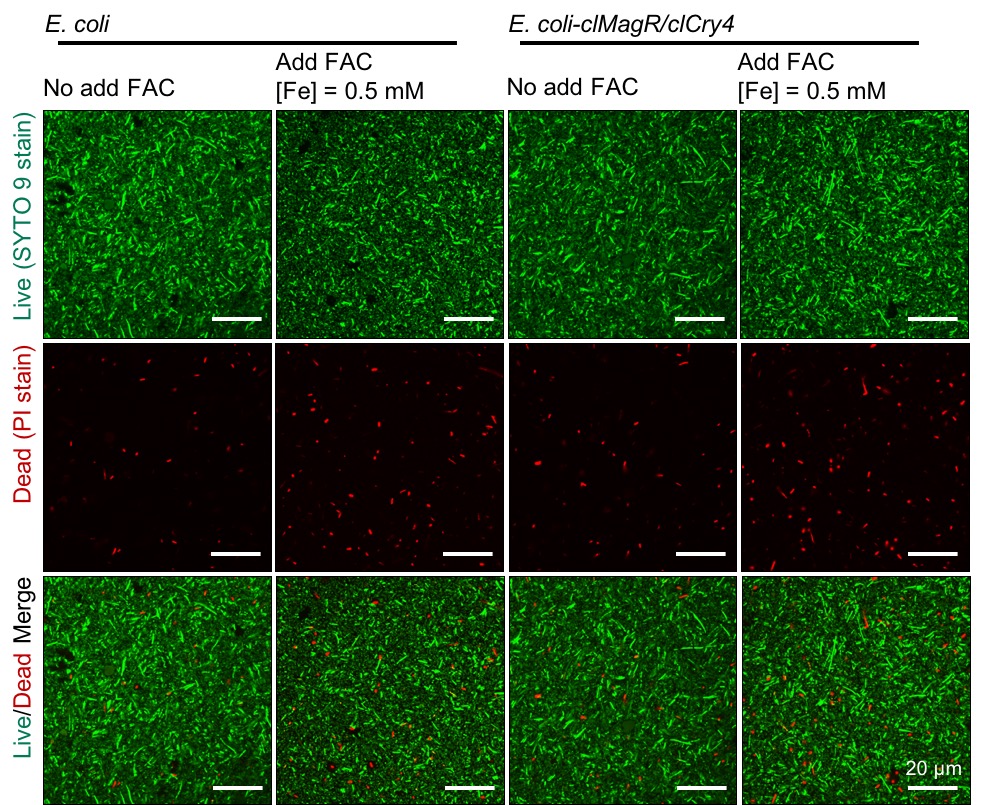


Live/Dead stain. Representative CLSM images of *E. coli*, stained with SYTO 9 and PI, showed a higher proportion of green fluorescence, corresponding to the live cell.

**
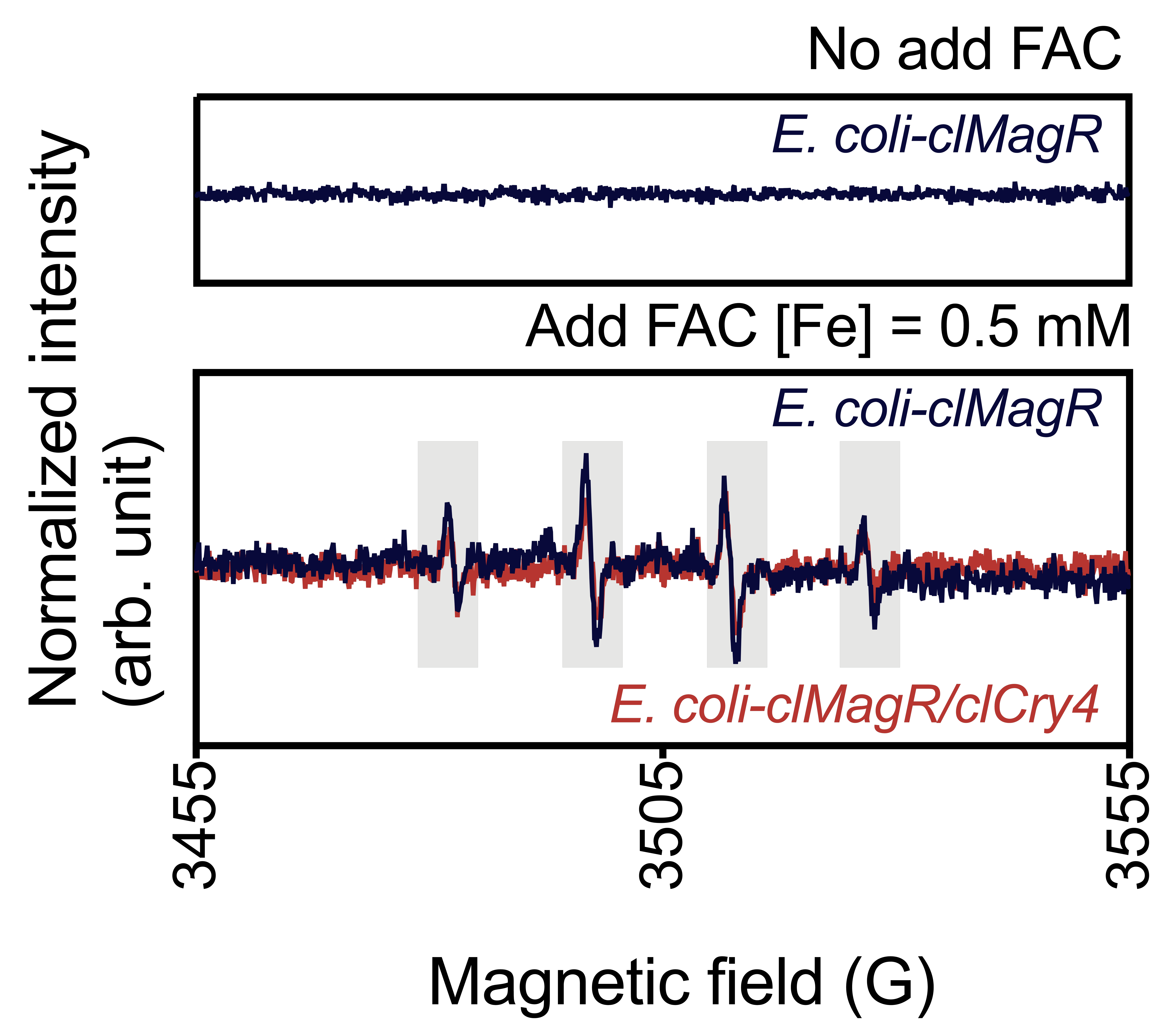
**


Hydroxyl radical generation monitored by ESR measurement.

**
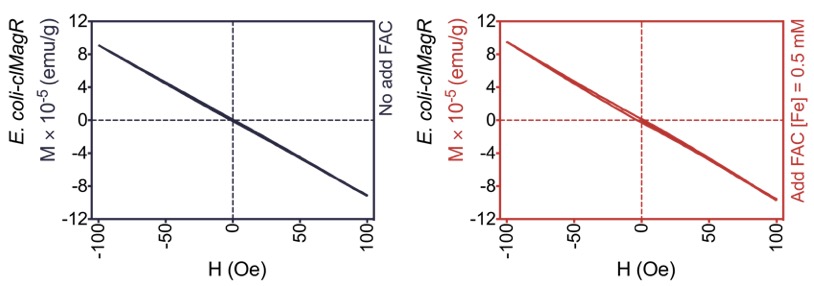
**


Hysteresis loops of the *E. coli* transfected with *clMagR*. It presented a diamagnetic property at the assay, even after exposure to FAC.


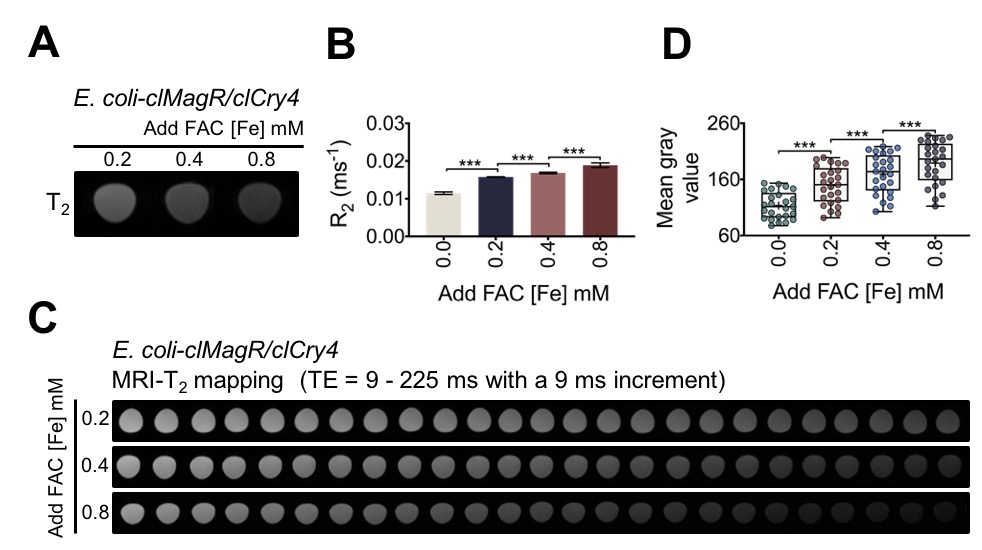


MRI-T_2_ analysis of the *E. coli* transfected with *clMagR/clCry4* under exogenous iron supply conditions. **(A)** MRI-T_2_ images show the signal intensity of the bacteria. The MRI T_2_ signal contrast turned more remarkable accompanied by iron concentration increased. **(B)** MRI statistical R_2_ values analysis of the bacteria. **(C,D)** Bacterial MRI-T_2_ mapping and the corresponding mean gray values analysis. Data were presented as mean ± SD (n = 3). For MRI R_2_ values, statistical differences were analyzed using the unpaired Student's t-test. For the mean gray value data, statistical significances were analyzed using two-way ANOVA followed by Bonferroni and Tukey’s HSD *post-hoc* test. ****P* < 0.001.


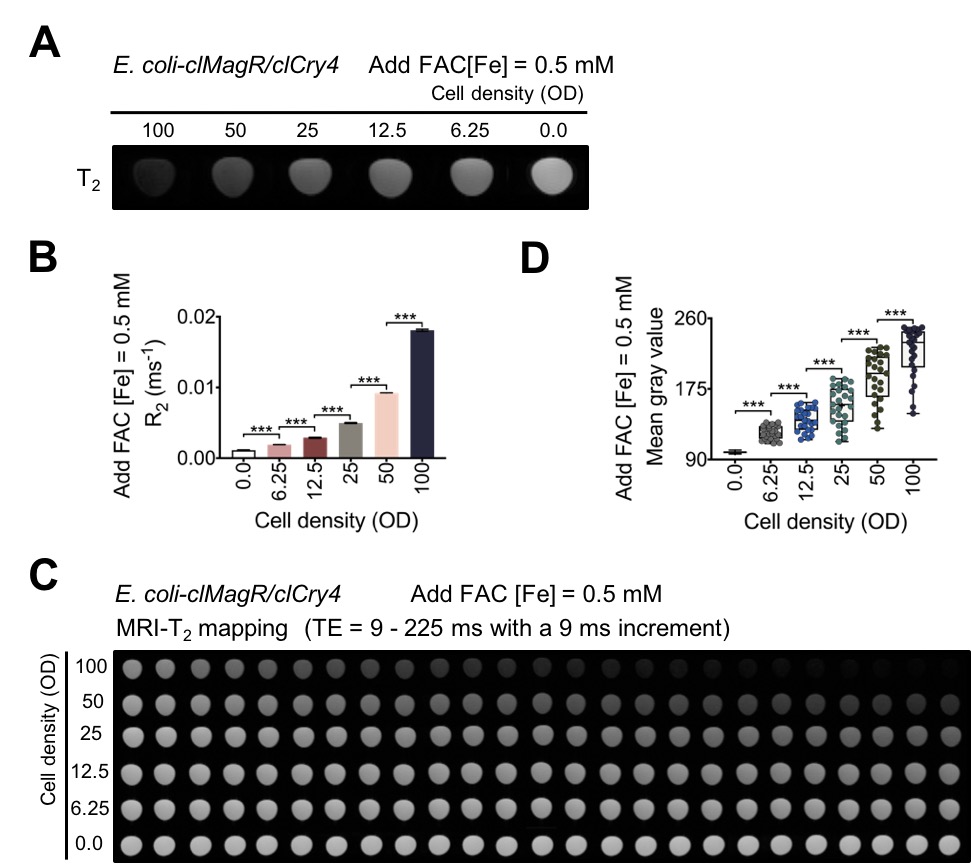


MRI-T_2_ analysis of the *E. coli* transfected with *clMagR/clCry4* under exogenous iron supply conditions. **(A)** MRI-T_2_ images show the signal intensity of the bacteria. The MRI T_2_ signal contrast turned more remarkable accompanied by cell density increased. **(B)** MRI statistical R_2_ values analysis of the bacteria. **(C,D)** Bacterial MRI-T_2_ mapping and the corresponding mean gray values analysis. Data were presented as mean ± SD (n = 3). For MRI R_2_ values, statistical differences were analyzed using the unpaired Student's t-test. For the mean gray value data, statistical significances were analyzed using two-way ANOVA followed by Bonferroni and Tukey’s HSD *post-hoc* test. ****P* < 0.001.


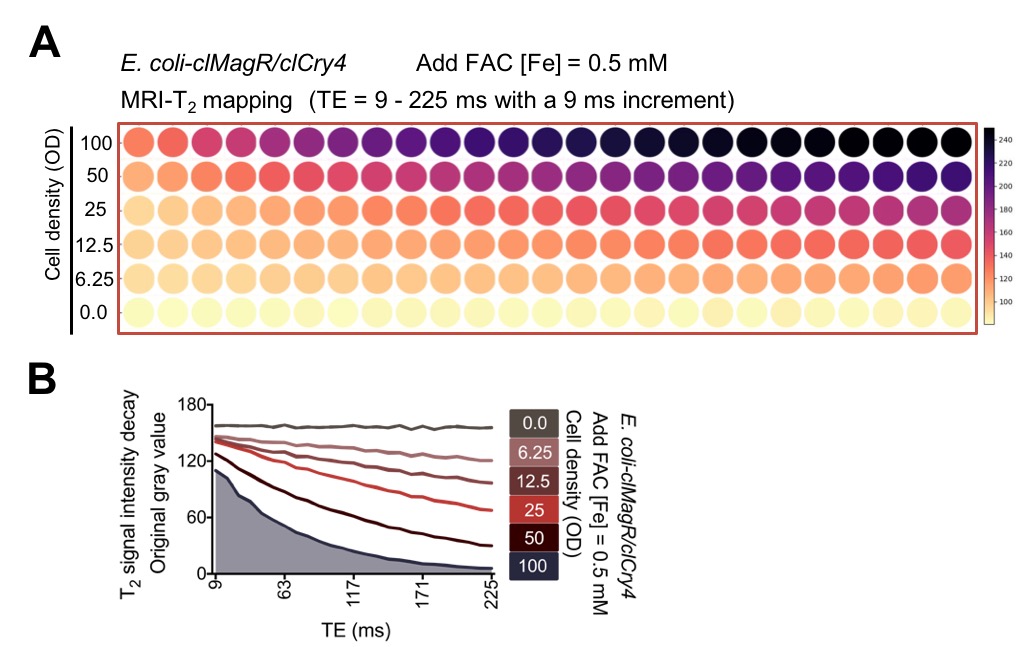


MRI-T_2_ analysis of the *E. coli* transfected with *clMagR/clCry4* under exogenous iron supply conditions. **(A)** Heat map indicating that *E. coli* transfected with *clMagR/clCry4* presented an enhanced contrast at gradient echo times compared to the control (OD 0.0, PBS). **(B)** Trendlines show the exponential decay of the experimental results: with the increase of cell density, the original gray value of the transfected *E. coli* showed as an exponential increase versus TE of on MRI-T_2_ mapping.


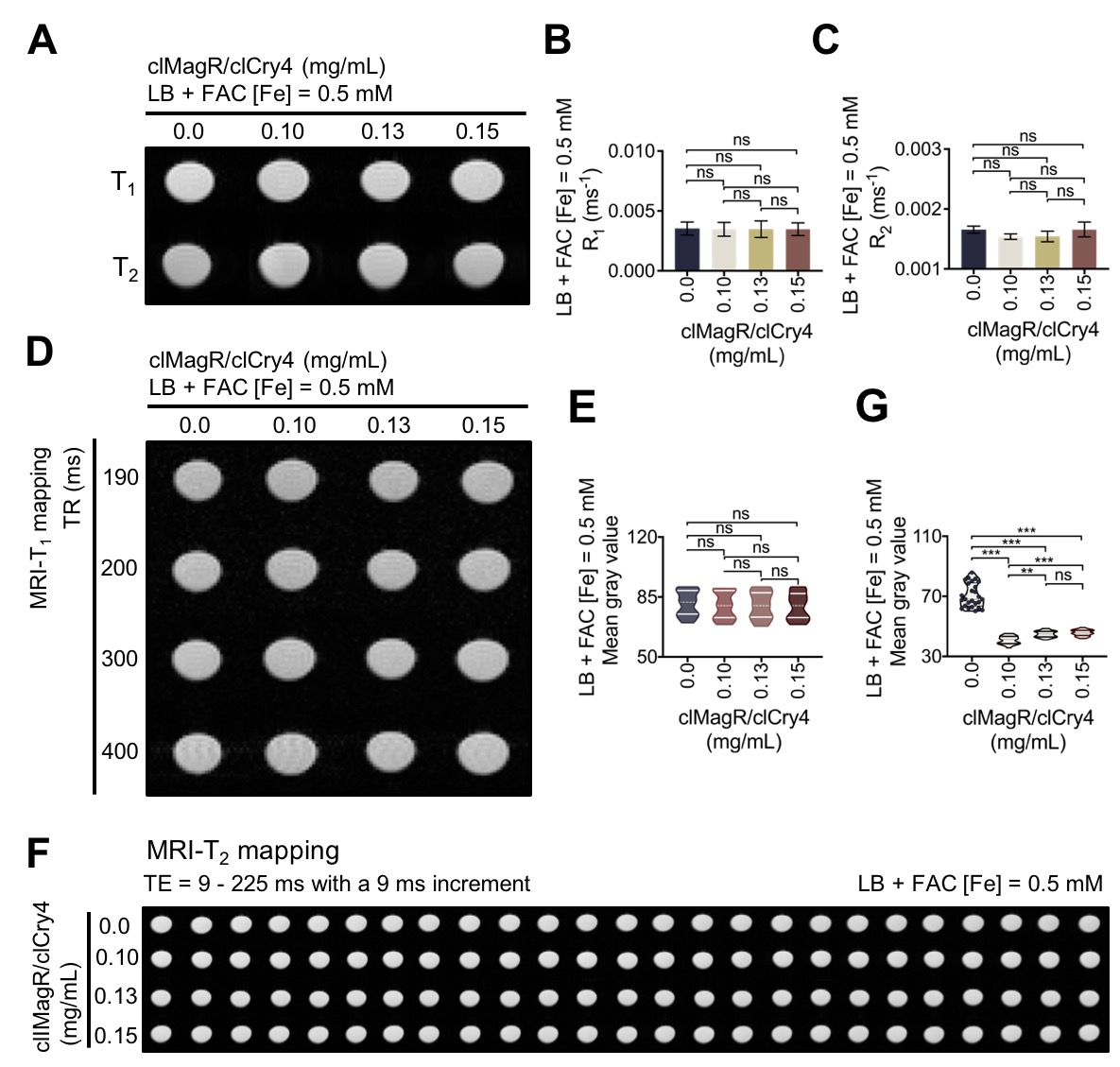


MRI analysis of the clMagR/clCry4 protein under exogenous iron supply conditions. **(A)** MRI-T_1_ and -T_2_ images show the signal intensity of the protein. Protein buffer solution was served as control (0 mg/mL). **(B,C)** Histogram representing the statistical values R_1_ and R_2_. **(D,E)** Protein MRI-T_1_ mapping and the corresponding mean gray values analysis. **(F,G)** Protein MRI-T_2_ mapping and the corresponding mean gray values analysis. Data were presented as mean ± SD (n = 3). For MRI R_2_ values, statistical differences were analyzed using the unpaired Student's t*-*test. For the mean gray value data, statistical significances were analyzed using two-way ANOVA followed by Bonferroni and Tukey’s HSD *post-hoc* test. ns, no significance (*P* > 0.05), ***P* < 0.01, ****P* < 0.001.


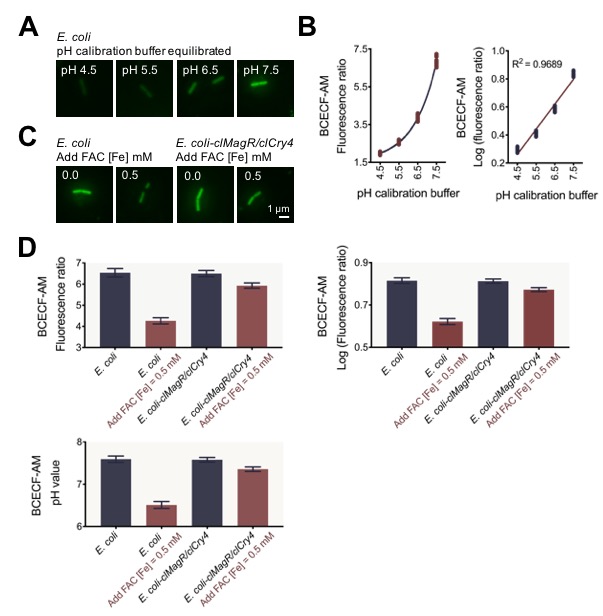


Intracellular pH detection. **(A)** pH-dependent fluorescence intensity of bacteria. Representative fluorescence microscopy images obtained from bacteria using BCECF-AM dye and pH calibration buffer (pH 4.5, 5.5, 6.5 and 7.5). **(B)** Calibration of the fluorescence ratio versus pH was performed by pH calibration buffer (solid circles indicated experimental values). Fluorescence emission of 535 nm was measured for excitations at 490 and 440 nm. The fit curve showing the fluorescence ratio (F_490_/F_440_) increases with increasing pH value. **(C)** Representative fluorescence microscopy images obtained from bacteria. **(D)** Histograms represented the experimental values obtained from bacteria, as determined by fluorescence ratio. Data were presented as mean ± SD (n = 25).


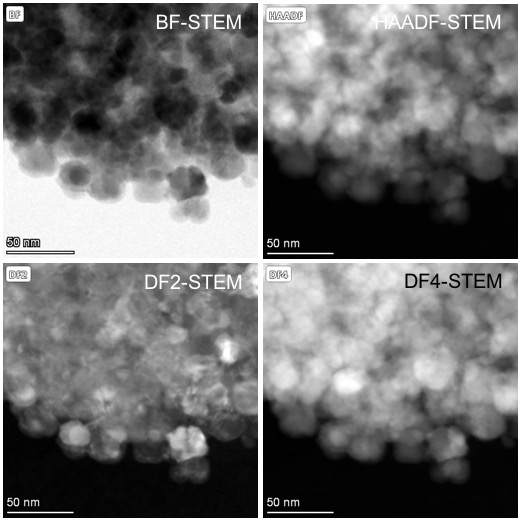


Electron microscope analysis. Morphology characteristics were observed by STEM micrographs.

**
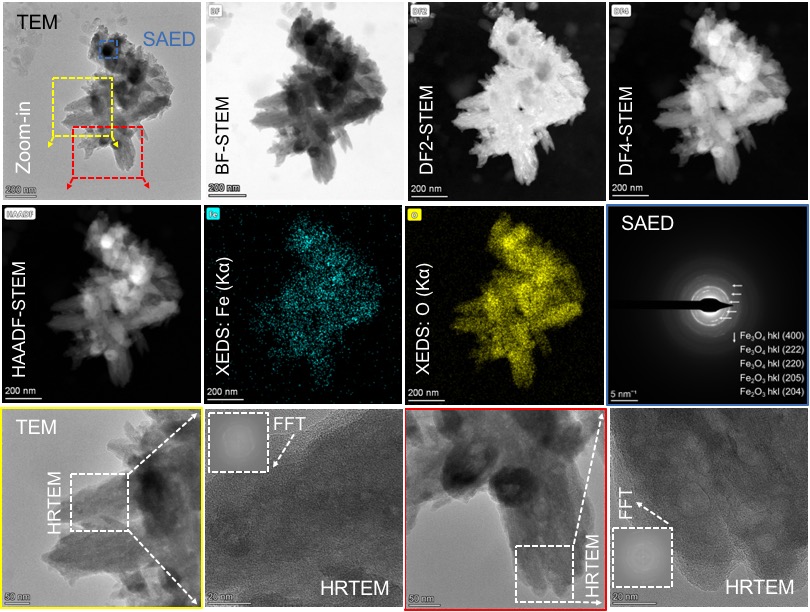
**

Electron microscope analysis. The tabular-like ferric (oxyhydr) oxides precursor phase with poorly crystalline was further confirmed by HRTEM and FFT studies.

| TABLE S1 Intracellular pH detection. | |
| --- | --- |
| Samples | pH values Mean ± SD (n = 25) |
| *E. coli* | 7.48 ± 0.02 |
| *E. coli-clMagR/clCry4* | 7.52 ± 0.01 |
| *E. coli*  Add FAC [Fe] = 0.5 mM | 6.24 ± 0.02 |
| *E. coli-clMagR/clCry4*  Add FAC [Fe] = 0.5 mM | 7.36 ± 0.02 |

**
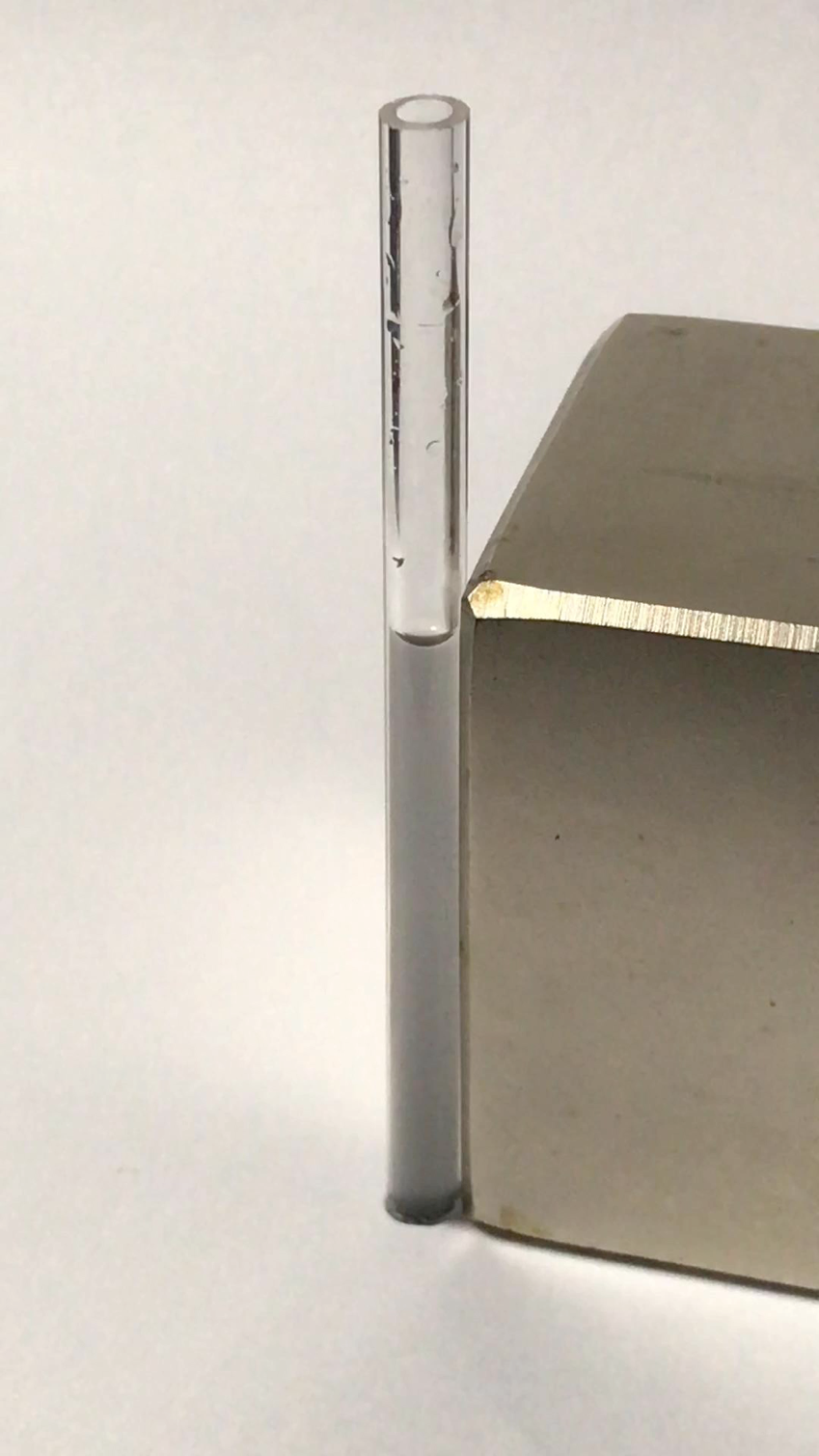
**

The particles obtained in the *E. coli* transfected with *clMagR/clCry4* migrate toward the NdFeB magnet. Here, the rectangular NdFeB magnet was adjoined to the particles suspension and magnetic flux density at the edge of the magnet was measured to be about 600 mT.
